# Predictors of Liver Fat and Stiffness in Non-Alcoholic Fatty Liver Disease (NAFLD) – an 11-Year Prospective Study

**DOI:** 10.1038/s41598-017-14706-0

**Published:** 2017-11-06

**Authors:** Susanna Lallukka, Sanja Sädevirta, Markus T. Kallio, Panu K. Luukkonen, You Zhou, Antti Hakkarainen, Nina Lundbom, Marju Orho-Melander, Hannele Yki-Järvinen

**Affiliations:** 1 Minerva Foundation Institute for Medical Research, Helsinki, Finland; 2 Department of Medicine, University of Helsinki, and Helsinki University Hospital, Helsinki, Finland; 3 Systems Immunity University Research Institute and Division of Infection and Immunity, School of Medicine, Cardiff University, Cardiff, United Kingdom; 4 HUS Medical Imaging Center, Helsinki University Hospital, Helsinki, Finland; 5 Department of Clinical Sciences, Diabetes and Endocrinology, University Hospital Malmö, Lund University, Malmö, Sweden

## Abstract

Liver fat can be non-invasively measured by proton magnetic resonance spectroscopy (^1^H-MRS) and fibrosis estimated as stiffness using transient elastography (FibroScan). There are no longitudinal data on changes in liver fat in Europids or on predictors of liver stiffness using these methods. We determined liver fat (^1^H-MRS) and clinical characteristics including features of insulin resistance at baseline and after a median follow-up period of 11.3 (range 7.3–13.4) years in 97 Finnish subjects. Liver stiffness was measured at 11.3 years. Liver fat content decreased by 5% (p < 0.05) over time. Values at baseline and 11.3 years were closely interrelated (*r* = 0.81, p < 0.001). Baseline liver fat (OR 1.32; 95%CI: 1.15–1.50) and change in BMI (OR 1.67; 95%CI: 1.24–2.25) were independent predictors of liver fat at 11.3 years (AUROC 0.90; 95%CI: 0.83–0.96). Baseline liver fat (AUROC 0.84; 95%CI: 0.76–0.92) predicted liver fat at 11.3 years more accurately than routinely available parameters (AUROC 0.76; 95%CI: 0.65–0.86, p = 0.02). At 11.3 years, 29% of the subjects had increased liver stiffness. Baseline liver fat (OR 2.17; 95%CI: 1.05–4.46) was an independent predictor of increased liver stiffness. These data show that liver fat is more important than the associated metabolic abnormalities as the predictor of future liver fat and fibrosis.

## Introduction

Non-alcoholic fatty liver disease (NAFLD) covers a range of conditions from simple steatosis (non-alcoholic fatty liver, NAFL) to non-alcoholic steatohepatitis (NASH) and fibrosis^1^. NAFL has been considered a benign and non-progressive condition^2^. Recent paired-biopsy studies in NAFLD patients have challenged this dogma by showing that NAFL can progress to NASH and fibrosis^3–5^, although the rate of progression is slow (1 stage over 14.3 years)^6^. It is unclear whether steatosis and the associated hypoxia and cell death around the central venous vein result in fibrosis via stellate cell activation^7,8^ or whether features of insulin resistance associated with ‘Obese/Metabolic’ NAFLD are of importance for fibrogenesis^9,10^. In support of the role of steatosis alone, patients with NAFLD due to genetic risk variants in patatin-like phospholipase domain-containing 3 (*PNPLA3*), transmembrane 6 superfamily member 2 (*TM6SF2)* and membrane bound O-acyltransferase domain containing 7 *(MBOAT7)* develop the full spectrum of NAFLD while they are neither insulin resistant nor at high risk of type 2 diabetes or cardiovascular diseases^11–14^.

The natural course of steatosis was recently characterized using proton magnetic resonance spectroscopy (^1^H-MRS), the state-of-the-art technique to quantify steatosis, in 565 Chinese subjects followed for 3.9 years^15^. Fatty liver developed in 14% of the subjects during the follow-up^15^. Kim *et al*. studied 76 obese youth of mixed ethnic origin for an average of 1.9 years and found liver fat measured by magnetic resonance imaging to remain unchanged during the follow-up^16^. Similar data are not available in Europid subjects.

Liver fibrosis can be non-invasively estimated using 1D ultrasonography transient elastography (TE; FibroScan, Echosens, Paris, France). The recent EASL-EASD-EASO Clinical Practice Guidelines for management of NAFLD and EASL-ALEH Clinical Practice Guidelines for evaluation of liver disease severity concluded that this technique is an acceptable non-invasive procedure for identification of cases at high risk of advanced fibrosis and cirrhosis^1,17^. Liver stiffness measurement (LSM) by TE predicts overall and liver-related mortality in NAFLD^18^ but we are not aware of longitudinal studies searching for predictors of increased liver stiffness.

In the present study, we examined the natural course of liver triglyceride content using^1^H-MRS in 97 Finnish subjects during an 11-year follow-up period, and determined which baseline factors predict NAFLD (liver triglyceride content exceeding 5.6%) and liver stiffness measured by TE. We were particularly interested to determine whether it is baseline liver fat or some feature of obesity/insulin resistance that best predicts liver stiffness.

## Study Subjects and Design

### Study subjects

We invited volunteers who had previously been participating in metabolic studies addressing liver fat content in our laboratory between years 1998 and 2004^19–24^. The subjects for the metabolic studies had been recruited based on newspaper advertisements and by contacting physicians in the Helsinki University Hospital region. Inclusion criteria at baseline were i) age 18 to 75 years, ii) no known acute or chronic disease except for obesity, hypertension, NAFLD or type 2 diabetes based on medical history, physical examination and standard laboratory tests (blood counts, serum creatinine, thyroid-stimulating hormone, electrolyte concentrations) and electrocardiogram, and iii) alcohol consumption less than 20 g per day in women and less than 30 g in men. The response rate was 73%. At follow-up, we examined 109 subjects of whom 12 were excluded because of excessive use of alcohol at the follow-up visit (n = 9), use of herbal medicinal products (n = 1) or cortisone (n = 1), or who underwent bariatric surgery (n = 1). The number of subjects studied was thus 97. Between the two study visits, the subjects received their usual treatment in the outpatient clinic if needed but did not participate in any intervention studies.

The study was conducted in accordance with the Declaration of Helsinki. Each participant provided written informed consent after being explained the nature and potential risks of the study. The ethics committee of the Helsinki University Hospital approved the study protocol.

### Study design

#### Baseline visit

At the baseline visit, medical history was obtained and a physical examination was performed. Fasting blood samples were taken for measurement of total blood counts and plasma creatinine, alanine aminotransferase (ALT), aspartate aminotransferase (AST), gamma-glutamyltransferase (GGT), high-density lipoprotein (HDL) and low-density lipoprotein (LDL) cholesterol, triglyceride, glucose, glycosylated hemoglobin A_1C_ (HbA_1C_), albumin, potassium, sodium, serum insulin and free fatty acid (FFA) concentrations. Homeostasis model assessment of insulin resistance (HOMA-IR) was calculated from the formula: fasting glucose (mmol/l) × fasting insulin (mU/l)/22.5^25^. The NAFLD fibrosis score was calculated based on knowledge of age, body mass index (BMI), impaired fasting glucose/diabetes status, concentrations of AST, ALT and albumin, and platelet count as described^26^. A pregnancy test in serum was performed in women of childbearing age. Proton magnetic resonance spectroscopy (^1^H-MRS) was used for measurement of liver fat content.

#### Follow-up visit

At this visit, medical history and physical examination were repeated. Fasting blood samples were taken for measurement of the same biochemical parameters and in the same laboratory as at baseline. In addition, antibodies against hepatitis A (HAVAbG and HAVAbM), B (HBcAb) and C (HCVAb), transferrin saturation, and anti-smooth muscle, anti-nuclear and anti-mitochondrial antibodies were measured. A pregnancy test in serum was performed in women of childbearing age. We also obtained blood samples for genotyping study subjects for NAFLD risk variants in *PNPLA3* at rs738409, *TM6SF2* at rs58542926 and *MBOAT7* at rs641738. Thereafter, a 2-hour oral glucose tolerance test (OGTT) was performed in non-diabetic subjects. Measurement of liver fat content by ^1^H-MRS was repeated. In addition, 92 subjects participated in a separate visit during which liver stiffness was measured using TE after an overnight fast. We used non-invasive imaging methods to measure liver steatosis and to estimate fibrosis as it was considered unethical to perform invasive liver biopsies without clinical indication in most of the study subjects.

## Results

### Liver fat content

**Figure 1:**
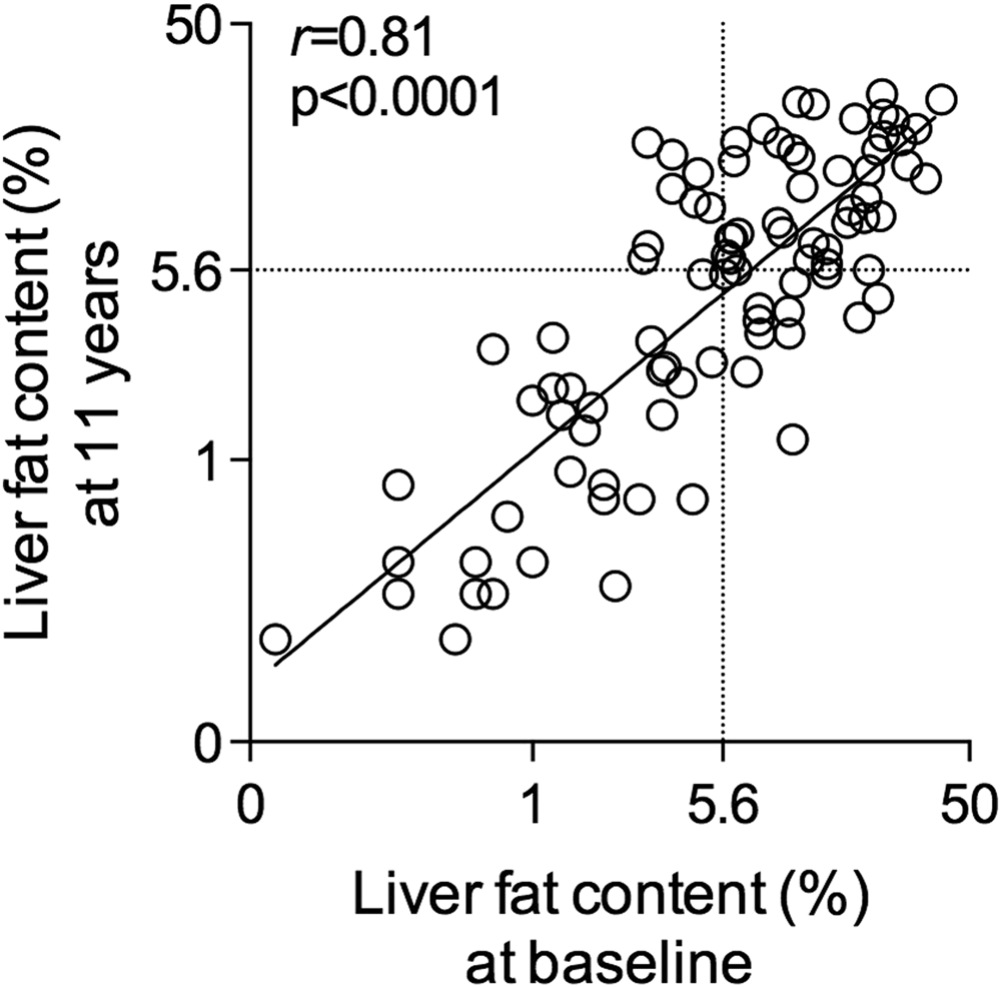
The relationship (Pearson’s correlation coefficient, *r*) between liver fat content measured by ^1^H-MRS at baseline and at 11 years.

Baseline characteristics of the study subjects are shown in Table 1. The median duration of follow-up was 11.3 and ranged from 7.3 to 13.4 years. Liver fat content decreased slightly by 5% from a median of 6.1% (25–75th percentile: 1.9–14.0%) at baseline to 5.8% (1.9–13.1%) at 11.3 years (p = 0.02). Individual values at baseline and at 11.3 years were highly interrelated (*r* = 0.81, p < 0.0001, Fig. 1). Of subjects without NAFLD at baseline, 79% remained free of NAFLD, and 73% of those with NAFLD at baseline still had NAFLD after the 11.3-year follow-up period.

**Table 1:** Characteristics of 97 study subjects and of those with low and increased liver stiffness at follow-up.

	All	Low liver stiffness (71%)		Increased liver stiffness (29%)	
	*Baseline*	*Baseline*	*Follow-up*	*Baseline*	*Follow-up*
Age (years)	44 ± 1	42 ± 1	53 ± 1^###^	46 ± 2	57 ± 2^###^
Gender (Men/Women)	46/51	23/29	—	11/10	—
Body composition					
Weight (kg)	84.8 ± 1.5	79.5 ± 1.6	82.5 ± 1.9^#^	86.7 ± 3.0*	88.9 ± 2.8
BMI (kg/m^2^)	29.0 ± 0.5	27.1 ± 0.5	28.1 ± 0.6^#^	29.6 ± 1.2*	30.4 ± 1.2
Waist circumference (cm)	99.0 ± 1.3	93.9 ± 1.4	93.4 ± 1.7	101.3 ± 2.8*	103.1 ± 2.8*
Body fat (%)	30.2 (22.2–35.8)	27.6 (20.6–34.8)	30.8 (24.7–37.8)^###^	30.2 (23.0–36.2)	32.4 (26.0–38.5)^##^
Liver fat (%)	6.1 (1.9–14.0)	4.1 (1.4–9.1)	3.5 (1.3–8.4)	10.8 (4.2–19.9)**	13.4 (4.2–17.3)**
Liver fat ≥5.56% (%)	55%	44%	40%	71%*	62%*
Blood pressure					
Systolic (mmHg)	125 ± 2	125 ± 2	141 ± 3^###^	127 ± 3	146 ± 3^##^
Diastolic (mmHg)	82 ± 1	80 ± 1	89 ± 2^###^	84 ± 2	90 ± 2^#^
Measures of glucose homeostasis					
fP-glucose (mmol/l)	5.8 (5.3–6.4)	5.6 (5.2–6.1)	5.4 (4.9–5.8)^###^	5.6 (5.1–6.3)	5.6 (4.9–6.4)
fS-insulin (mU/l)	7.0 (5.0–12.0)	6.5 (4.0–9.0)	5.4 (3.1–10.3)	9.0 (5.5–14.8)*	11.1 (6.1–12.6)*
HOMA-IR	2.0 (1.2–3.5)	1.5 (1.1–2.4)	1.3 (0.7–2.4)	2.6 (1.4–3.7)	2.9 (1.6–3.3)*
HbA_1C_ (%)	5.7 (5.3–6.1)	5.5 (5.3–6.0)	5.6 (5.3–6.0)	5.9 (5.4–6.1)	6.0 (5.5–6.7)^#,^*
Type 2 diabetes (%)	22%	15%	19%	24%	43%*
Glucose-lowering medication (%)	14%	8%	23%^#^	14%	24%
Insulin (%)	8%	6%	10%	10%	14%
Lipids					
fP-Triglycerides (mmol/l)	1.3 (0.9–1.9)	1.3 (0.9–1.7)	1.1 (0.8–1.5)	1.5 (1.0–2.6)	1.3 (0.9–2.2)
fP-HDL cholesterol (mmol/l)	1.3 (1.1–1.5)	1.3 (1.1–1.6)	1.3 (1.2–1.6)^#^	1.2 (1.1–1.4)	1.2 (1.1–1.5)
fP-LDL cholesterol (mmol/l)	3.1 (2.4–3.8)	3.0 (2.3–3.8)	2.9 (2.4–3.6)	3.0 (2.4–3.5)	3.0 (2.1–3.8)
fS-FFA (μmol/l)	689 ± 24	656 ± 29	500 ± 25^###^	692 ± 46	536 ± 44^#^
Lipid medication (%)	9%	4%	35%^###^	14%	14%
Liver enzymes and function tests					
P-ALT (U/l)	28 (20–48)	27 (19–49)	28 (21–37)	36 (22–48)	40 (21–51)
P-AST (U/l)	27 (22–38)	27 (22–38)	29 (25–36)	28 (25–40)	33 (28–50)*
P-GGT (U/l)	25 (15–46)	18 (14–41)	23 (16–43)	30 (19–60)	31 (19–60)
AST/ALT ratio	0.91 (0.69–1.10)	0.95 (0.66–1.12)	1.04 (0.86–1.21)^#^	0.90 (0.74–1.09)	1.03 (0.94–1.22)^#^
P-Albumin (g/l)	41.1 ± 0.4	41.3 ± 0.5	38.8 ± 0.3^###^	41.9 ± 1.0	38.7 ± 0.5
B-platelet count (10^9^/l)	234 (194–269)	231 (202–272)	235 (204–277)	241 (194–270)	231 (203–258)
B-leukocyte count (10^9^/l)	5.8 ± 0.2	5.5 ± 0.2	5.5 ± 0.2	6.5 ± 0.3*	6.7 ± 0.3**
Risk variants for NAFLD					
PNPLA3 (148^II^/148^IM^/148^MM^)	54/33/10	30/15/7	—	12/8/1	—
TM6SF2 (CC/CT/TT)	74/17/1	39/10/0	—	17/3/1	—
MBOAT7 (CC/CT/TT)	35/43/14	17/26/6	—	8/7/6	—

Data are shown as number, percent, mean ± standard error of mean or median (the 25^th^–75^th^ percentiles). ^#^p < 0.05, ^##^p < 0.005^, ###^p < 0.0005 as compared to baseline within the group. *p < 0.05, **p < 0.005, ***p < 0.0005 as compared to the ‘Low liver stiffness’ group at the same visit. Abbreviations: ALT, alanine aminotransferase; AST, aspartate aminotransferase; B, blood; BMI, body mass index; FFA, free fatty acid; fP, fasting plasma; fS, fasting serum; GGT, gamma-glutamyl transferase; HbA_1C_, glycosylated hemoglobin A_1C_; HDL, high-density lipoprotein; HOMA-IR, homeostasis model assessment of insulin resistance; LDL, low-density lipoprotein; MBOAT7, membrane bound O-acyltransferase domain containing 7; NAFLD, non-alcoholic fatty liver disease; P, plasma; PNPLA3, patatin-like phospholipase domain-containing protein 3; TM6SF2, transmembrane 6 superfamily member 2.

In univariate analysis, of baseline parameters, measures of obesity, concentrations of fasting glucose, insulin, triglycerides, HDL cholesterol and liver enzymes, and liver fat content predicted liver fat content at 11.3 years (Table 2).

**Table 2:** Univariate analyses for baseline predictors of liver fat and increased stiffness at follow-up.

	Liver fat content (n = 91)		Liver stiffness (n = 73)	
	*r*	p-value	OR (95% CI)^a^	p-value
Age (years)	0.352	0.001	1.06 (0.99–1.12)	0.09
Male gender	−0.156	0.13	1.39 (0.50–3.83)	0.53
Body composition				
Liver fat content (log)	0.809	<0.001	2.48 (1.27–4.84)	0.01
Waist circumference (cm)	0.481	<0.001	1.06 (1.01–1.11)	0.02
Waist-to-Hip Ratio (per SD)	0.359	0.001	2.04 (1.10–3.79)	0.03
BMI (kg/m^2^)	0.412	<0.001	1.15 (1.02–1.30)	0.03
Weight (kg)	0.306	0.003	1.05 (1.00–1.10)	0.03
Body fat percent (log)	0.422	<0.001	1.26 (0.76–2.08)	0.37
Measures of glucose homeostasis				
HOMA-IR (log)	0.560	<0.001	1.70 (0.95–3.04)	0.07
fS-insulin (log)	0.512	<0.001	1.96 (1.06–3.63)	0.03
HbA_1C_ (log)	0.281	0.007	1.25 (0.74–2.11)	0.41
fP-glucose (log)	0.259	0.01	1.04 (0.62–1.73)	0.89
Blood leukocyte count (log)	0.253	0.02	2.12 (1.19–3.78)	0.01
Liver enzymes				
P-ALT (log)	0.448	<0.001	1.14 (0.69–1.89)	0.61
AST/ALT ratio (log)	−0.419	<0.001	0.93 (0.60–1.61)	0.93
P-GGT (log)	0.375	0.001	1.40 (0.82–2.40)	0.21
P-AST (log)	0.271	0.009	1.18 (0.73–1.92)	0.49
Lipids				
fP-Triglycerides (log)	0.371	<0.001	1.47 (0.88–2.44)	0.13
fP-HDL cholesterol (log)	−0.347	0.001	0.59 (0.34–1.05)	0.07
fP-LDL cholesterol (mmol/L)	0.046	0.67	0.91 (0.51–1.63)	0.75
fS-FFA (μmol/L)	0.197	0.07	1.00 (1.00–1.00)	0.50
Risk variants for NAFLD				
PNPLA3 (148^II^/148^IM^/148^MM^)	0.150	0.16	1.02 (0.37–2.85)^b^	0.73
TM6SF2 (CC/CT/TT)	−0.001	0.99	0.92 (0.25–3.34)^b^	0.90
MBOAT7 (CC/CT/TT)	0.056	0.61	1.30 (0.63–2.68)	0.48
Number of risk alleles	0.129	0.25	1.15 (0.73–1.81)	0.54

^a^OR is calculated per unit, number or if logarithmic transformed per standard deviation (SD).

^b^148IM and 148MM/CT and TT groups are combined.

Abbreviations: ALT, alanine aminotransferase; AST, aspartate aminotransferase; BMI, body mass index; CI, confidence interval; FFA, free fatty acids; fP, fasting plasma; fS, fasting serum; GGT, gamma-glutamyl transferase; HbA_1c_, glycosylated hemoglobin A_1c_; HDL, high-density lipoprotein; HOMA-IR, homeostasis model assessment of insulin resistance; LDL, low-density lipoprotein; MBOAT7, membrane bound O-acyltransferase domain containing 7; OR, odds ratio; P, plasma; PNPLA3, patatin-like phospholipase domain-containing protein 3; *r*, Pearson’s correlation coefficient; TM6SF2, transmembrane 6 superfamily member 2.

**Figure 2:**
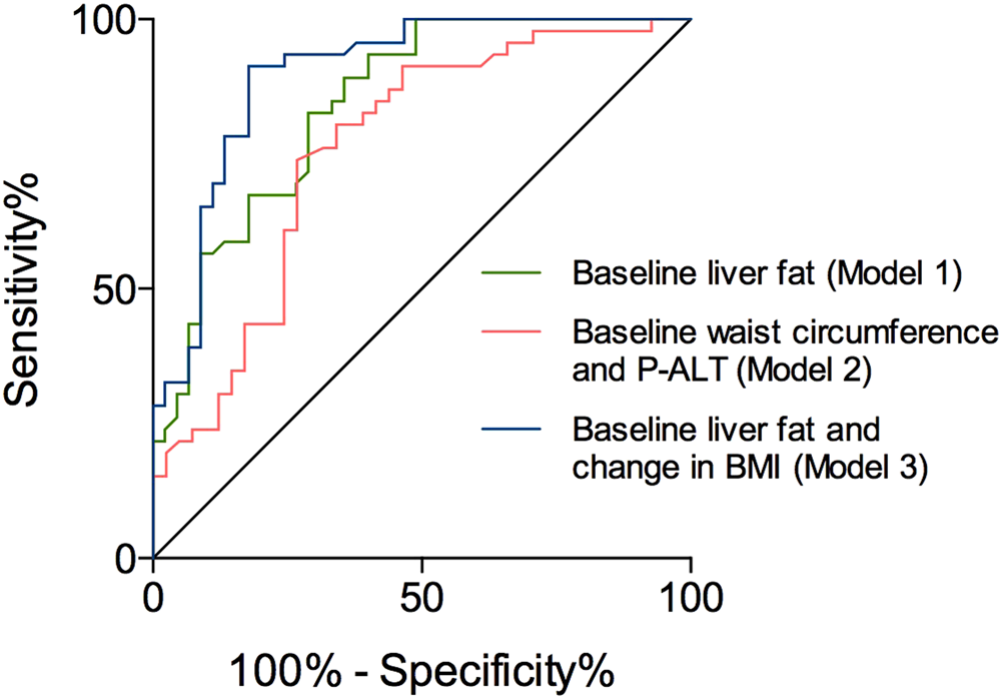
The receiver operating characteristic (ROC) curves of models to predict NAFLD (liver fat ≥5.56%) at follow-up. Models included significant predictors from univariate analyses: baseline age, BMI, waist circumference, fasting plasma triglyceride, blood leukocyte count, HOMA-IR, plasma ALT and liver fat content, and changes in waist circumference, BMI, HbA_1C_ and blood leukocyte count. *Model 1 (green) included all significant baseline predictors*: baseline liver fat content was independent predictor of NAFLD at follow-up with AUROC of 0.84 (95% CI: 0.76-0.92), p < 0.0001. *Model 2 (red) included all significant baseline predictors except for liver fat content*: baseline waist circumference and plasma ALT were independent predictors of NAFLD at follow-up with AUROC of 0.76 (95% CI: 0.65-0.86), p < 0.0001. *Model 3 (blue) included all significant baseline predictors and changes during a follow-up period*: baseline liver fat content and change in BMI remained independent predictors of NAFLD at follow-up with AUROC of 0.90 (95% CI: 0.83-0.96), p < 0.0001. *Comparison between models using the method of DeLong et al*.: Model 1 (p = 0.02) and Model 3 (p = 0.006) were significantly better than Model 2 to predict NAFLD at follow-up. The difference between Model 1 and Model 3 was not significant (p = 0.07). Abbreviations: ALT, alanine aminotransferase; AUROC, area under the receiver operating characteristic curve; BMI, body mass index; CI, confidence interval; HbA_1C_, glycosylated hemoglobin A_1C_; HOMA-IR, homeostasis model assessment of insulin resistance; NAFLD, non-alcoholic fatty liver disease..

In a multiple binary logistic regression model, liver fat content remained the only independent predictor of NAFLD at 11.3 years (odds ratio: 1.22, 95% confidence interval (CI): 1.11–1.34, p < 0.001; Table 3). To determine how well routinely available clinical variables at baseline predict liver fat at 11 years, we included in another logistic regression model significantly associated baseline variables other than liver fat. In this model, baseline waist circumference and plasma ALT were independent predictors of NAFLD at 11.3 years (Table 3). The area under the receiver operating characteristic curve (AUROC) of the first model including baseline liver fat (0.84, 95% CI: 0.76–0.92, p < 0.0001 for this AUROC) was significantly greater than that of the latter model including only routinely available parameters (0.76, 95% CI: 0.65–0.86, p < 0.0001 for this AUROC and p = 0.02 for comparison between the models) (Fig. 2).

**Table 3:** Multiple binary logistic regression models to predict NAFLD and increased liver stiffness.

	AUROC	B	SE	P-value	OR (95% CI)
**Models to predict NAFLD**					
‘*All baseline parameters*’ (*Model 1*)*	0.84 (0.76–0.92), p < 0.0001				
Baseline liver fat (per 1%)		0.198	0.049	<0.001	1.22 (1.11–1.34)
Constant		−1.519	0.421	<0.001	0.22
‘*Baseline parameters except for liver fat*’ *(Model 2)* ^#^	0.76 (0.65–0.86), p < 0.0001				
Baseline waist circumference (per 1 cm)		0.065	0.023	0.005	1.07 (1.02–1.12)
Baseline P-ALT (per 10 U/L)		0.27	0.125	0.03	1.31 (1.03–1.67)
Constant		−7.375	2.314	0.001	0.001
‘*All baseline parameters and all changes except for liver fat*’ *(Model 3)*°	0.90 (0.83–0.96), p < 0.0001				
Baseline liver fat (per 1%)		0.274	0.067	<0.001	1.32 (1.15–1.50)
Change in BMI (per 1 kg/m^2^)		0.513	0.152	0.001	1.67 (1.24–2.25)
Constant		−2.768	0.678	<0.001	0.063
Model to predict liver stiffness**					
Baseline liver fat (log, per SD)	0.74 (0.61–0.87), p = 0.002	0.773	0.368	0.04	2.17 (1.05–4.46)
Baseline leukocyte count (log, per SD)		0.598	0.327	0.07	1.82 (0.96–3.45)
Constant		−0.985	0.313	0.002	0.373

*Model 1 (‘*All baseline parameters*’) included baseline age, BMI, waist circumference, plasma ALT, fasting plasma triglyceride, blood leukocyte count, HOMA-IR and baseline liver fat content.

^#^Model 2 (‘*Baseline parameters except for liver fat*’) included all parameters in Model 1 except for baseline liver fat.

°Model 3 (‘*All baseline parameters and changes except for liver fat*’) including parameters in Model 1 and changes in waist circumference, BMI, HbA_1c_ and blood leukocyte count during 11 years.

**Model predicting liver stiffness included baseline age, weight, waist-to-hip ratio, fasting serum insulin (log), fasting plasma HDL cholesterol (log), blood leukocyte count (log) and liver fat content (log).

ALT, alanine aminotransferase; AUROC, area under the receiver operating characteristic curve; B, coefficient; BMI, body mass index; CI, confidence interval; HbA_1C_, glycosylated hemoglobin A_1C_; HDL, high-density lipoprotein; HOMA-IR, homeostasis model assessment of insulin resistance; log, logarithmic transformed; NAFLD, non-alcoholic fatty liver disease; OR, odds ratio; SD, standard deviation; SE, standard error.

The relationship between changes in various parameters and liver fat content at follow-up are shown in Supplementary Table 1. When changes were included in the model also containing baseline parameters, of changes only that  in BMI remained an independent predictor of NAFLD at 11.3 years (Table 3). The AUROC of this model was 0.90 (95% CI: 0.83–0.96, p < 0.0001), which was not significantly better than that containing only baseline liver fat content (Fig. 2). The relationship between change in liver fat and changes in BMI, fasting triglycerides, HOMA-IR and ALT concentrations are depicted in Supplementary Fig. 1.

We also created a multiple linear regression equation to allow prediction of liver fat content at follow-up with the help of baseline liver fat (Model 1 in Supplementary Table 2). Exclusion of baseline liver fat worsened the model considerably (Model 2 in Supplementary Table 2). In multiple linear regression analysis, which included significant baseline parameters and changes during follow-up, baseline liver fat and changes in BMI and HbA_1C_ remained significant independent predictors and explained 74% (p < 0.001) of the variation in liver fat content at 11 years (Model 3 in Supplementary Table 2).

### Liver stiffness and NAFLD fibrosis score

At follow-up, 29% of the subjects had increased liver stiffness (Table 1). In these subjects, the median LSM was 10.4 kPa (25–75^th^ percentile: 9.5–14.3 kPa) measured with the M probe (n = 11) and 10.3 kPa (25–75^th^ percentile: 7.9–12.3 kPa) measured with the XL probe (n = 10) at follow-up. In subjects with low liver stiffness at follow-up, the median LSM with the M probe was 5.4 kPa (25–75^th^ percentile: 4.3–6.8 kPa; n = 42) and with the XL probe 5.8 kPa (25–75^th^ percentile: 4.2–6.6 kPa; n = 10). Subjects who had increased liver stiffness at follow-up were significantly more obese, had wider waist circumferences, and had higher serum insulin concentrations and blood leukocyte counts at baseline than subjects whose liver stiffness remained below the cut-off values (Table 1). Baseline liver fat content was significantly higher in subjects with (10.6%, 4.0–20.0) than without (4.1%, 1.4–9.7, p < 0.001) increased stiffness. The NAFLD fibrosis score increased significantly during 11 years from a median of −2.202 (−2.713 to −1.294) at baseline to −0.989 (−1.776 to −0.095, p < 0.001) at follow-up. Aging explained 32% of this change. The increase in NAFLD fibrosis score during 11 years was significant even if age was kept constant (−1.379 (−2.152 to −0.510) at follow-up, p < 0.001 for change).

Baseline parameters, which were significantly associated with increased liver stiffness at follow-up (Table 2), were entered in multiple binary logistic regression analysis. Baseline liver fat content remained an independent predictor of increased liver stiffness at follow-up (Table 3). The AUROC of this model was 0.74 (95% CI: 0.61–0.87, p = 0.002).

### Liver biopsies

Liver biopsies were obtained in seven patients with clinical indication. The histological features of these biopsies are listed in Table 4.

**Table 4:** Liver stiffness and histological assessment of liver biopsies (n = 7).

	Liver stiffness (kPa)	Steatosis (%)	Ballooning (yes/no)	Lobular inflammation (yes/no)	Stage of fibrosis
1.	6.6^a^	60	Yes	Yes	1
2.	7.0^a^	10	No	No	0
3.	10.2^a^	80	Yes	Yes	1
4.	10.4^a^	60	Yes	Yes	2
5.	10.4^b^	30	Yes	Yes	2
6.	28.4^b^	40	No	No	2
7.	72.0^b^	30	No	No	4

^a^Measured using the XL probe.

^b^Measured using the M probe.

## Discussion

In this longitudinal study, we measured liver fat content using ^1^H-MRS at an interval of around 11 years. Although mean liver fat content decreased significantly by 5%, baseline and follow-up values were highly interrelated. Of subjects without NAFLD, 79% remained free of NAFLD while NAFLD persisted in 73% of those with NAFLD at baseline. Baseline liver fat content and the change in BMI were the best predictors of liver fat at follow-up in multivariate analyses. At 11 years, 29% of the subjects had increased liver stiffness. At baseline, these subjects were more obese, had wider waist circumferences, and had higher liver fat content and blood leukocyte counts than subjects with more elastic livers. In multivariate analysis, baseline liver fat content remained the only independent predictor of liver stiffness.

Liver fat at baseline and follow-up were significantly correlated (*r* = 0.81) in the present study. This correlation coefficient was identical to that observed in 76 obese youth followed for 1.9 years^16^. In the study in Hong-Kong, the correlation coefficient between baseline and follow-up liver fat values was 0.39 in subjects without and 0.50 in those with NAFLD at baseline^15^. Consistent with the current data, a study using ultrasound to diagnose NAFLD found 81% of 147 subjects without steatosis at baseline to remain free of NAFLD, while 64% of 66 subjects with NAFLD at baseline still had NAFLD after a 7-year period of follow-up^27^. Although individual values were closely correlated at baseline and follow-up, liver fat content decreased slightly and significantly. This is in line with paired-biopsy studies, in which steatosis grade significantly decreased while fibrosis progressed during 3.2 years of follow-up of 103 subjects of unspecified ethnic origin^28^, during 6.4 years of follow-up of 132 Italian subjects^29^ and during 13.7 years of follow-up of 68 Swedes^30^. In 52 Chinese subjects with NAFLD, steatosis grade increased significantly while fibrosis stage remained stable during 3 years^4^.

In addition to baseline liver fat, several measures of obesity and concentrations of fasting glucose, insulin, triglycerides and HDL cholesterol predicted liver fat measured by ^1^H-MRS at follow-up in univariate analyses. These data, which to our knowledge are the first in Europid adults, resemble those in Chinese^15^. In the latter study, subjects who developed NAFLD diagnosed by ^1^H-MRS were more obese and had higher glucose and triglyceride concentrations and lower concentrations of HDL cholesterol than those who did not develop a fatty liver^15^. Most studies assessing steatosis by ultrasound have been performed in Asians^31–34^. In these studies, baseline obesity^31–33^, age^32,33^, components of metabolic syndrome^32,33^ and serum ferritin^34^ predicted NAFLD. The change in BMI was the only significant predictor of the change in liver fat during 11 years in the present study. Similarly, weight gain was associated with development and weight loss with remission of NAFLD diagnosed by ultrasound in 213 Israeli subjects^27^.

To our knowledge, this is the first longitudinal study identifying predictors of increased liver stiffness in NAFLD. We did not perform LSM at baseline, which is a limitation. However, at least when judged from the NAFLD fibrosis score, fibrosis did significantly worsen during the follow-up, even when normalized for age. Liver fat content was an independent predictor of liver stiffness at the end of the 11-year follow-up period. The data are consistent with paired-biopsy studies showing that steatosis predicts fibrosis^3,5,6^.

The ability of liver fat but not metabolic features to predict stiffness 11 years later may give hints of the pathogenesis of fibrosis. It is well established that patients with NAFLD due to the genetic risk variants in *PNPLA3* ^35^, *TM6SF2* ^36^ and *MBOAT7* ^13^ are at risk of fibrosis but these patients are neither more obese nor insulin resistant compared to non-carriers of the variants^37–39^. This would suggest that steatosis, the common denominator between ‘Obese/Metabolic’ NAFLD and NAFLD due to the risk variants, rather than insulin resistance could facilitate the development of fibrosis. In liver lobules, fat accumulates and hepatocytes undergo cell death (ballooning) around the central vein^40^. This process leads to activation of hepatic stellate cells and perisinusoidal deposition of collagen again starting from the central venous area^8,40^. This pathophysiology could explain why steatosis rather than the associated metabolic features predicts fibrosis, although measurement of steatosis is neither necessary nor sufficient to detect fibrosis.

Strengths of our study include a long follow-up period and use of a state-of-the-art quantitative method to measure of liver fat with ^1^H-MRS. The data are also the first to describe predictors of increased liver stiffness associated with NAFLD. An important limitation of our study is that the study subjects were not selected from a population-based sample but were recruited by newspaper advertisements and by contacting local physicians and thus selection bias may exist. Our study is also underpowered to detect effects of the genetic risk variants on liver fat content. Furthermore, liver biopsies were only available from subjects in whom a liver biopsy was considered to be clinically justified.

We conclude that liver fat decreases slightly although NAFLD status remains markedly stable over an 11-years. Baseline liver fat content is the best predictor of both liver fat and stiffness during 11 years of follow-up while routinely available clinical and biochemical parameters are significantly less accurate predictors. These data support the view that steatosis rather than the associated metabolic abnormalities is important in the pathogenesis of fibrosis.

## Methods

### Measurement of liver fat using proton magnetic resonance spectroscopy (^1^H-MRS)

The liver fat content was measured using three generations of 1.5 Tesla clinical scanners (Magnetom Vision, Sonata and Avanto, Siemens Healthcare Diagnostics, Erlangen, Germany). The intensity differences arising from various acquisition parameters and localization techniques had to be normalized. T1-weighted high-resolution magnetic resonance imaging scans were collected using a standard ^1^H body coil. The ^1^H-MRS voxel of interest (8 to 27 cm^3^) was carefully located within the right lobe of the liver avoiding subcutaneous fat, large vessels, bile ducts and the gall bladder. Localization was performed using the STEAM sequence with echo time (TE)/mixing time (TM)/repetition time (TR) of 20/30/3000 ms and 32 acquisitions for Vision measurements and the PRESS sequence with TE/TR of 30/3000 ms and 16 acquisitions for Sonata and Avanto measurements. Subjects were breathing normally during the data collection. All spectra were analyzed with the MRUI/jMRUI software using VARPRO/AMARES (available at www.mrui.uab.es/mrui/). The intensities of the peaks resonating from the protons of water, and protons of methylene (CH_2_)_n-2_ groups in the fatty acid chains were determined using line-shape fitting with prior knowledge. Signal intensities were corrected for T1 and T2 relaxation using the equation I_m_ = I_0_ exp(−TE/T2)*[1−exp(−(TR−TM−0.5TE)/T1)]*exp(−TM/T1) for Vision data and the equation I_m_ = I_0_ exp(−TE/T2) for Sonata and Avanto data. T1 of 600 ms^41^ and 300 ms^42^ and experimentally determined T2 of 46 ms and 58 ms were used for water and fat, respectively. Liver fat content was expressed as a ratio of signal from methylene group to total signal of methylene and water. Liver fat content was converted from signal ratio to a weight fraction, applying method validated by Longo *et al*.^43^ and Szczepaniak *et al*.^44^. The following experimentally determined factors were used: i) the ratio of the number of lipid protons in the fitted (CH_2_)_n-2_ signal to the total number of lipid protons is 0.6332^45^; ii) proton densities of fat and water are 111 and 111 mol/l, respectively; iii) 1 g liver tissue contains 711 mg water; iv) densities of the liver tissue, fat in the liver, and water are 1.051 g/ml, 0.900 g/ml, and 1.000 g/ml; respectively. The measurement has been validated against histologically determined lipid content^46^ and against estimates of fatty degeneration or infiltration by x-ray computer-assisted tomography^21^. A physicist who was unaware of any of the clinical data analyzed all spectra. NAFLD was defined as liver fat >5.56% by ^1^H-MRS as in the Dallas Heart Study^44^.

### Liver stiffness measurement (LSM) using transient elastography (TE)

LSM was used as a non-invasive test estimating liver fibrosis and was measured when the patients were lying supine with their right arm in maximal abduction using TE (FibroScan, Echosens, Paris, France). Two experienced physicians (S.L. and P.K.L.) performed LSMs using the same protocol. The tip of the probe transducer was covered with gel and placed on the skin at the level of the right lobe of the liver. The depth of the measurement was 25–65 mm below the skin surface using the M probe and 35–75 mm using the XL probe. LSM was first performed with the M probe. If obesity prevented adequate measurement, the XL probe was used. We used the cut-off values of 8.7 kPa with the M probe and 7.2 kPa with the XL probe for clinically significant stage 3–4 fibrosis, as recommended^47^. Subjects in whom 10 acquisitions were successful and interquartile range divided by median (IQR/median) was less than 0.3 were included in analyses. LSM failed with both probes in eight subjects. The results are expressed as the median value of ten successful measurements in kilopascal (kPa).

### Liver biopsies

When clinically indicated, a percutaneous liver biopsy was obtained under ultrasound guidance using a 16 G BioPince Full Core Biopsy instrument (Argon Medical Devices, Athens, TX). All biopsies were > 20 mm in length and sent to the pathologist for histological assessment. Histology was analyzed by an experienced liver pathologist in a blinded fashion according to the criteria proposed by Brunt *et al*.^40^.

### Genotyping of *PNPLA3*, *TM6SF2* and *MBOAT7* risk variants

Genomic DNA was extracted from whole blood using the Autopure LS (Qiagen, Hilden, Germany). All three single-nucleotide polymorphisms *(*SNP; *PNPLA3* at rs738409, C>G/I148M; *TM6SF2* at rs58542926, C>T/E167K; and *MBOAT7* at rs641738, C>T) were genotyped by TaqMan PCR method (Applied Biosystems, Foster City, CA) according to manufacturer’s instructions. Post-PCR allelic discrimination was carried out measuring allele-specific fluorescence on an ABI Prism Sequence Detection System ABI 7900HT (Applied Biosystems). The success rate for genotyping was >95% for all three SNPs and the genotypes of all three SNPs were in Hardy–Weinberg equilibrium.

### Analytical procedures

Fasting plasma glucose was measured using a hexokinase method on an autoanalyser (Roche Diagnostics Hitachi 917, Hitachi Ltd., Tokyo, Japan). Serum insulin concentration was determined by time-resolved fluoroimmunoassay using Insulin Kit (AUTOdelfia, Wallac, Turku, Finland). HbA_1C_ was measured by high-pressure liquid chromatography using a fully automated Glycosylated Hemoglobin Analyzer System (BioRad, Richmond, CA). Plasma total and HDL cholesterol and triglyceride concentrations were measured with respective enzymatic kits from Roche Diagnostics using an autoanalyzer (Roche Diagnostics Hitachi 917, Hitachi Ltd., Tokyo, Japan). Serum FFA concentration was measured by an enzymatic colorimetric assay (NEFA-HR(2), Wako Chemicals GmbH, Neuss, Germany) using a Konelab 60i analyzer (Thermo Electron Corporation, Vantaa, Finland). Plasma ALT, AST, GGT and creatinine concentrations were determined as recommended by the European Committee for Clinical Laboratory Standards.

### Other measurements

Body weight was recorded to the nearest 0.1 kg using a calibrated digital scale (Soehnle, Monilaite-Dayton, Finland) with barefoot subjects wearing light indoor clothing. Height and circumferences of waist and hip were recorded to the nearest 0.5 cm using a non-stretchable tape. BMI was defined as weight/height^2^ (kg/m^2^). Waist circumference was measured midway between the lower rib margin and the iliac crest and hip circumference over the greater trochanters. The percentage of body fat was determined using bioelectric impedance analysis (BioElectrical Impedance Analyzer System model #BIA-101A, RJL Systems, Detroit, MI). Blood pressure was measured in a sitting position after a minimum of 15 minutes of acclimatization and before blood sampling using an automatic sphygmomanometer (OMRON M7, Omron Healthcare Co. Ltd., Kyoto, Japan).

### Statistical analyses

Distribution of continuous variables was tested for normality using the Shapiro-Wilk’s normality test. Normally distributed data are shown as mean ± standard error of mean and non-normally distributed data as median followed by the 25^th^ and 75^th^ percentiles. Changes during follow-up within the study groups were analyzed using the paired t-test or Wilcoxon’s matched pairs test, and the groups were compared with the unpaired t-test or with the Mann-Whitney’s test, as appropriate. For categorical variables, we used Fisher’s exact test or McNemar’s test. We applied logarithmic transformation for non-normally distributed data for Pearson’s correlation coefficient and linear regression analyses.

Linear and binary logistic regression analyses were used to identify predictors of liver fat and stiffness. Variables predicting liver fat and stiffness at a significance level of <0.05 in univariate analyses were entered in multiple linear or backward logistic regression analyses as appropriate. If variables were measures of the same biological process (weight/BMI/body fat percent, waist/hip/waist-to-hip ratio, glucose/HbA_1C_/insulin/HOMA-IR), we only included the one most closely associated with outcome of interest. The area under the receiver operating characteristic (ROC) curve (AUROC) of the logistic regression models was used to compare models by the method of DeLong *et al*.^48^. GraphPad Prism version 6.00 for Mac (GraphPad Software, San Diego, CA), IBM SPSS Statistics 24.0 for Mac (IBM SPSS, Chicago, IL) and the’pROC’ package in R (http://www.R-project.org/) were used to perform the statistical analyses. A two-sided p-value of less than 0.05 was considered statistically significant.

### Data Availability

The data analyzed during the current study are available from the corresponding author on reasonable request.

## Electronic supplementary material

Supplementary material
