# Supplementary material for: Predictors of Liver Fat and Stiffness in Non-Alcoholic Fatty Liver Disease (NAFLD) – an 11-Year Prospective Study
Source: Sci Rep. 2017 Nov 6;7:14561. doi: 10.1038/s41598-017-14706-0 (PMC5674024; doi:10.1038/s41598-017-14706-0)
Supplement: Supplementary file 1 — Supplementary material [file 41598_2017_14706_MOESM1_ESM.pdf]

SUPPLEMENTARY MATERIAL

PREDICTORS OF LIVER FAT AND STIFFNESS IN NON-ALCOHOLIC FATTY LIVER DISEASE (NAFLD) – AN 11-YEAR PROSPECTIVE STUDY

Susanna Lallukka[1,2], Sanja Sädevirta[1,2], Markus T. Kallio[1,2], Panu K. Luukkonen[1,2], You Zhou[1,3], Antti Hakkarainen[4], Nina Lundbom[4], Marju Orho-Melander[5], Hannele Yki-Järvinen[1,2]

[1]Minerva Foundation Institute for Medical Research, Helsinki, Finland; [2]Department of Medicine, University of Helsinki, and Helsinki University Hospital, Helsinki, Finland; [3]Systems Immunity University Research Institute and Division of Infection and Immunity, School of Medicine, Cardiff University, Cardiff, United Kingdom; [4]HUS Medical Imaging Center, Helsinki University Hospital, Helsinki, Finland; [5]Department of Clinical Sciences, Diabetes and Endocrinology, University Hospital Malmö, Lund University, Malmö, Sweden

Supplementary Table 1. Pearson's correlation coefficients between changes in clinical and biochemical characteristics during a follow-up and liver fat content at 11 years.

	r	p-value
Body composition		
Change in liver fat content (log)	0.348	0.001
Change in waist circumference (cm)	0.311	0.003
Change in BMI (kg/m^2)	0.242	0.021
Change in body weight (kg)	0.209	0.046
Change in waist-to-hip ratio	0.186	0.086
Change in body fat (log)	-0.135	0.20
Measures of glucose homeostasis		
Change in HbA_{1C} (log)	0.284	0.007
Change in HOMA-IR (log)	0.268	0.019
Change in fS-insulin (log)	0.242	0.034
Change in fP-glucose (log)	0.023	0.83
Liver enzymes		
Change in P-ALT (log)	0.330	0.001
Change in P-AST (log)	-0.020	0.85
Change in AST/ALT ratio (log)	-0.010	0.93
Change in P-GGT (log)	0.161	0.16
Lipids		
Change in fP-HDL cholesterol (log)	0.016	0.88
Change in fP-LDL cholesterol (mmol/L)	-0.112	0.30
Change in fP-Triglycerides (log)	0.050	0.64
Change in fS-FFA (mmol/L)	0.108	0.34
Change in blood leukocyte count (log)	0.233	0.026

Abbreviations: ALT, alanine aminotransferase; AST, aspartate aminotransferase; BMI, body mass index; FFA, free fatty acid; fP, fasting plasma; fS, fasting serum; GGT, gamma-glutamyl transferase; HbA_{1C}, glycosylated hemoglobin A_{1C}; HDL, high-density lipoprotein; HOMA-IR, homeostasis model assessment of insulin resistance; LDL, low-density lipoprotein; P, plasma; *r*, correlation coefficient.

**Supplementary Table 2.** Multiple linear regression models to predict liver fat content at 11.3 years					
	Adjusted R^2	B	SE	P-value	Beta
*'All baseline parameters' (Model 1)**	0.65, p<0.001				
Baseline liver fat (log)		0.823	0.065	<0.001	0.809
Constant		0.047	0.059	0.424	
'Baseline parameters except for liver fat' (Model 2)[#]	0.41, p<0.001				
Baseline HOMA-IR (log)		0.703	0.174	<0.001	0.390
Baseline P-ALT (log)		0.529	0.196	0.008	0.256
Baseline age (years)		0.016	0.005	0.002	0.273
Constant		-1.100	0.364	0.003	
'All baseline parameters and changes except for liver fat' (Model 3)°	0.74, p<0.001				
Baseline liver fat (log)		0.797	0.058	<0.001	0.784
Change in BMI (kg)		0.062	0.012	<0.001	0.283
Change in HbA_{1C} (fold-change, log)		2.475	0.821	0.003	0.173
Constant		0.062	0.012	0.82	0.173

*Model 1 ('All baselineparameters') included baseline age, BMI, waist circumference, P-ALT (log), fP-triglyceride (log), blood leukocyte count (log), HOMA-IR (log) and liver fat content (log).

[#]Model 2 ('Baseline parameters except for liver fat') included parameters in Model 1 except for baseline liver fat content.

°Model 3 ('All baseline and changes except for liver fat') included parameters in Model 1 and changes in BMI, waist circumference, HbA_{1C}, (fold-change, log) and blood leukocyte count (fold-change, log).

ALT, alanine aminotransferase; B, unstandardized coefficient; Beta, standardized coefficient; BMI, body mass index; HbA_{1C}, glycosylated hemoglobin A_{1C}; HOMA-IR, homeostasis model assessment of insulin resistance; log, logarithmic transformed; P, plasma; R^2, coefficient of determination; SE, standard error for unstandardized coefficient.

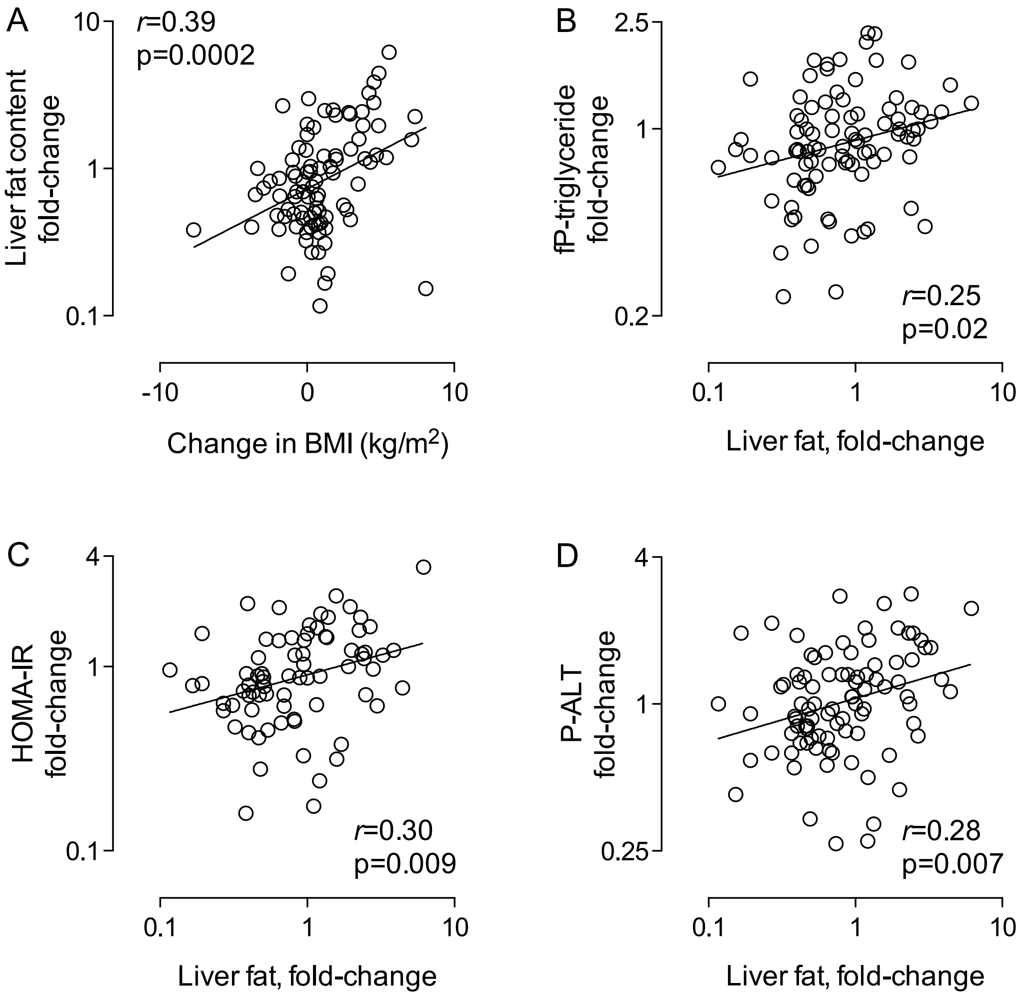

Supplementary Figure 1. The relationships (Pearson's correlation coefficients, *r*) between change in liver fat content (log, fold-change) measured by ^{1}H-MRS and changes in (A) BMI, (B) fP-triglycerides (log, fold-change), (C) HOMA-IR (log, fold-change) and (D) P-ALT (log, fold-change). Abbreviations: ALT, alanine aminotransferase; BMI, body mass index; fP, fasting plasma; HOMA-IR, homeostasis model assessment of insulin resistance; ^{1}H-MRS, proton magnetic resonance spectroscopy; P, plasma.
